# Emergencies in obese patients: a narrative review

**DOI:** 10.1186/s44158-021-00019-2

**Published:** 2021-11-13

**Authors:** Ida Di Giacinto, Martina Guarnera, Clelia Esposito, Stefano Falcetta, Gerardo Cortese, Giuseppe Pascarella, Massimiliano Sorbello, Rita Cataldo

**Affiliations:** 1 Unit of Anesthesia and Intensive Care, Mazzoni Hospital, Ascoli Piceno, Italy; 2 Department of Anesthesia and Intensive Care, Azienda Ospedaliero-Universitaria Sant’Orsola-Malpighi - Alma Mater Studiorum, Bologna, Italy; 3 Department of Anesthesia and Intensive Care, Ospedali dei Colli, Naples, Italy; 4 Department of Anesthesia and Intensive Care, Clinica di Anestesia e Rianimazione Ospedali Riuniti Ancona, Ancona, Italy; 5 Department of Anesthesia and Intensive Care, AOU Città della salute e della scienza Torino, Turin, Italy; 6 Department of Anesthesia and Intensive Care, Università Campus Bio-Medico, Via Alvaro del Portillo, 200 Rome, Italy; 7 Department of Anesthesia and Intensive Care, AOU Policlinico San Marco University Hospital, Catania, Italy

**Keywords:** Resuscitation, Obesity, Emergency, Cardiac arrest, Trauma

## Abstract

Obesity is associated to an increased risk of morbidity and mortality due to respiratory, cardiovascular, metabolic, and neoplastic diseases. The aim of this narrative review is to assess the physio-pathological characteristics of obese patients and how they influence the clinical approach during different emergency settings, including cardiopulmonary resuscitation. A literature search for published manuscripts regarding emergency and obesity across MEDLINE, EMBASE, and Cochrane Central was performed including records till January 1, 2021. Increasing incidence of obesity causes growth in emergency maneuvers dealing with airway management, vascular accesses, and drug treatment due to both pharmacokinetic and pharmacodynamic alterations. Furthermore, instrumental diagnostics and in/out-hospital transport may represent further pitfalls. Therefore, people with severe obesity may be seriously disadvantaged in emergency health care settings, and this condition is enhanced during the COVID-19 pandemic, when obesity was stated as one of the most frequent comorbidity. Emergency in critical obese patients turns out to be an intellectual, procedural, and technical challenge. Organization and anticipation based on the understanding of the physiopathology related to obesity are very important for the physician to be mentally and physically ready to face the associated issues.

## Introduction

Obese people are characterized by an increased risk of morbidity and mortality and an association between obesity and an increased risk of sudden cardiac death has also been recognized [1–3].

Even a subgroup of obese people known as “metabolically healthy,” which do not suffer from comorbidities associated with obesity, has been found to have an increased risk of cardiovascular diseases, stroke, and heart failure [4].

Despite all these possible disorders, from the literature emerges the phenomenon of the obesity paradox [5], that is a better outcome of out-of-hospital post-cardiac-arrest obese patients [6, 7] as well as better survival in case of myocardial infarction, unstable angina, atrial fibrillation, and heart failure compared with normal-weight or underweight patients [8–10]. Several hypotheses have been put forward to explain the phenomenon: first of all, the greater metabolic reserve of individuals with obesity. Secondly, due to the presence of numerous comorbidities, obese patients are more likely to follow appropriate drug therapy [11]. In addition, obese patients often suffer from obstructive sleep apnea (OSA), which exposes them to intermittent periods of hypoxemia predisposing them to systemic protective preconditioning [7]. Obesity is also characterized by an attenuated response of the renin-angiotensin-aldosterone system as well as reduced production of atrial BNP, resulting in the delayed progression towards a symptomatic state of congestive heart failure [12].

The increasing incidence of obesity worldwide is causing an increase in emergency procedures and resuscitation maneuvers to be performed in obese patients. The current guidelines (Advanced Life Support of the European Resuscitation Council, Advanced Cardiovascular Life Support of the American Heart Association) cite the patient with obesity as a “circumstance or special patient” whose resuscitation treatment does not differ from that of the normal-weight adult patient, recognizing the challenges related to the treatment of these patients without providing specific recommendations [13–15].

Since there are no defined guidelines for obese patients in an emergency, the aim of this narrative review is to assess the physiopathological characteristics of obese patients and how they influence the clinical approach during different emergency settings, including cardiopulmonary resuscitation.

## Search strategy

A literature search for published manuscript regarding emergency and obesity across MEDLINE (via PubMed), EMBASE, and Cochrane Central was performed combining the terms “obese,” “obesity,” “airway,” “emergency,” “trauma,” “cardiac arrest,” “cardiopulmonary resuscitation,” and “CPR.” Randomized controlled trials, observational studies, guidelines, letters to the editor, and case reports published till January 1, 2021, were included in the research strategy.

Search results were limited to publications in English; abstracts from conferences and commentaries were excluded. In the case of a guideline written by the same society or author group and underwent multiple publications, only the latest version was included in the results. All manuscripts underwent title and abstract screening for relevance to the aims of this review; potentially eligible articles were retrieved for full-text review. Screening of all reference lists of relevant studies was also performed, in order to identify any missing publications. Searches; title, abstract, and reference screening; and study selection were performed independently by three investigators (M.G., C.E., S.F.); discrepancies were resolved through consensus by a fourth investigator (G.C.).

## Practical tasks for the emergency management of obese patients

### Vascular access

Ensuring venous access is a priority in an emergency scenario but can be very difficult and may require numerous venipunctures that predispose the patient to thrombosis and catheter-related infections. The easiest veins to cannulate are those of the antecubital fossa of the arm, if possible with the pressure of a sphygmomanometer rather than a tourniquet.

If no peripheral venous access can be found, placement of central venous access must be considered in spite of obesity-related factors such as the presence of abundant adipose tissue at the access site, which can impede the identification of usual anatomical landmarks; frequently, obese patients are unable to tolerate the Trendelenburg position required for the maneuver [16].

The use of ultrasound in finding the venous, peripheral, and central accesses is mandatory to increase the chances of success and reduce the duration of the procedure and the risk of complications [17].

In this regard, in obese patients, the cephalic vein at the mid-arm may be easily visualized using ultrasound. This vein may be sufficiently deep and stable to be punctured under ultrasound guidance to insert long peripheral cannulas or “short midline” catheters which may constitute stable access for at least 24 h [18].

Alternatives to peripheral and central venous accesses include the intraosseous route, but once again, obesity can present anatomic barriers to successful placement. Kehrl et al. demonstrated that a standard 25-mm intraosseous needle may not be long enough for patients with a body mass index (BMI) > 43 kg/m^2^, and an extended 45-mm needle should be used if available [19].

### Airway management

A patient with obesity presents an increased complexity in airway management [20] related mainly to difficult oxygenation. Endotracheal intubation may be more challenging because of anatomical alterations: the patient with obesity can be difficult to ventilate and oxygenate, with a “cannot ventilate, cannot oxygenate” scenario occurring in the worst cases. In emergencies, therefore, even more than in elections, it is essential to be prepared for a difficult airway.

Many factors can influence the prediction and management of a difficult airway: [1] the operator’s skills and experience, operative timing, and device and drug availability [2]; the patient’s condition such as the level of urgency, state of consciousness and cooperation, gastric content, trauma, and positioning [21, 22]. In addition to the standard difficult airway indexes (Mallampati score, inter-incisive distance, head extension, thyromental distance, edentulism, or presence of fixed teeth or dentures), which are easy to assess even in an emergency if the patient is at least minimally alert and collaborative, there are several specific scores: the circumference of the neck > 41 cm in women and > 43 cm in men and the waist-to-hip ratio > 0.9 [23]. A STOP-Bang score ≥ 5 is highly suggestive of severe OSA and involves a greater risk of ventilation and difficult intubation, while the MACOCHA score is a valid assessment in case of an intensive care therapy scenario [24, 25].

Guarantee oxygenation in obese patients is a primary issue. If an airway management is needed, it is therefore imperative to provide the patient with adequate preoxygenation at positive pressure up to EtO2 > 90 mmHg with a facial mask, continuous positive airway pressure (CPAP) mask, or high-flow nasal cannula (HFNC) throughout the pre-intubation phase in the ramped or reverse Trendelenburg position [22]. The nasal oxygenation during an effort to secure a tube (NODESAT) technique with a nasal cannula or HFNC can be very useful during airway apneic instrumentation [26]. Endotracheal intubation should follow the “first pass success” approach, that is, the first attempt must be made in the best possible conditions to optimize the chances of success: ramped position and video laryngoscopy or standard laryngoscopy with the tube already mounted on the introducer or stylet, depending on the operator skills are advisable.

If endotracheal intubation fails, ventilation with supraglottic devices or return to the facial mask is imperative. In case proceed without delay to cricothyrotomy after having identified the cricothyroid membrane, using ultrasound can improve the chance of success [27]. Modified rapid sequence induction and intubation (mRSII) does not have a mandatory indication in the patient with obesity; rather, the same indications apply as in non-obese patients (full stomach, symptomatic esophageal reflux, pregnancy, diabetes with gastroparesis) with the addition, however, of any previous bariatric surgery.

The mRSII strategy no longer involves the use of succinylcholine; instead, rocuronium is given at a dose of 1–1.3 mg/kg of lean body weight (LBW), with a propofol bolus in emergency or urgency conditions. Cardiovascular impairment must be prevented by filling fluids, if possible, and/or vasoactive drug administration. Alternatively, the use of ketamine is allowed.

Numerous studies have been conducted on the ventilation of obese patients in the operating room and in intensive care whose findings are also applicable in the context of emergencies [28].

Protective ventilation helps to reduce the inflammatory response caused by damage to the alveolar-capillary barrier. Low tidal volume (Vt 6–8 ml/kg according to ideal body weight [IBW] or predicted body weight) avoids ventilation-induced lung injury,; moderate positive end-expiratory pressure (PEEP) (< 10 cmH_2_O) prevents atelectotrauma (the cyclic opening and closing of the alveoli during every respiratory act), and a driving pressure (difference between plateau pressure and PEEP) < 16 cmH_2_O avoids barotrauma. It is advisable to use recruiting maneuvers only as a rescue in case of desaturation [29, 30].

### Emergency transport

Transport of the patient with pathological obesity can be extremely difficult. Depending on the patient’s size and the local circumstances, numerous skilled rescuers may be necessary to lift and transfer the patient to the ambulance. If the patient is found inside a confined space, firefighters are necessary to facilitate transport outside. Stretchers or sheets intended for the transport of heavy weights are available on the market. The helicopter transfer of obese patients could be possible according to their body weight, time/distance of transport, fuel required, and crew member. In-hospital transport can also be problematic: staff must be adequately trained in patient transport, and the hospital must be equipped with suitable devices [31–33].

### Diagnostic procedures

Ultrasonography is the standard technique in emergency diagnostics. However, performing an ultrasound examination in a patient with obesity is technically complex due to the hypo-echogenicity of the adipose tissue and the distance between the skin and the target organs. The use of 2-Mhz probes is recommended, as this allows to reach a greater depth at the expense, however, of spatial resolution.

Due to the patient’s size, visualization of the entire body region may not be possible with a single standard X-ray. Some images, such as a lateral view of the cervical spine, are also difficult to interpret. Computerized tomography (CT) and magnetic resonance and magnetic resonance imaging (MRI) devices have size and weight restrictions. Consequently, multiple scans may be necessary, with increased timing, radiation exposure, and the risk of motion artifacts [34].

No clear evidence exist about the first-choice diagnostic exam to perform in this setting, although a lot depends on clinicians’ expertise and device availability.

## Complex emergency scenarios in obese patients

### Acute respiratory failure

Obese people are at higher risk to have acute respiratory failure (ARF) in the presence of other conditions like chronic obstructive pulmonary disease (COPD), asthma, infections, cardiac diseases, and surgery. This predisposition could be strongly related to an underestimated condition, which is called obesity hypoventilation syndrome (OHS) [35].

OHS is a chronic disease associated with respiratory and cardiometabolic impairments leading to a decrease in the ability to perform normal daily activities along with a higher risk of hospitalization and death [36]. It is defined as a combination of obesity (BMI > 30 kg/m^2^), PacO2 > 45 mmHg, daytime hypercapnia, and disordered breathing during sleep [37].

Continuous airway pressure (CPAP) is recommended as the first-line treatment to stable ambulatory patients with OHS and coexistent severe obstructive sleep apnea (OSA) [38].

Non-invasive ventilation (NIV) in intensive care unit (ICU) setting is widely used for the management of acute hypercapnic respiratory failure in these patients, whereas oxygen therapy alone or with the use of CPAP may not resolve hypoventilation [37]. NIV reduces the respiratory load, increases the minute volume for a given breathing effort, and provides ventilation during central apneic events.

Although so far there is a lack of evidence to guide NIV in the population with obesity presenting with ARF, some settings have been underlined: positive end-expiratory airway pressure (PEEP) ranging from 8 to 12 mmHg with minimal pressure support (8–10 cmH_2_O) is commonly required. The respiratory rate should be set to 2–3 breaths/min below the resting respiratory rate. Moreover, prolonging inspiratory time (*Ti*) increases VT, and a more upright position may help in better ventilation. Finally, volume assured mode may be considered when very high inspiratory pressure is required [39, 40].

However, since NIV can have failure rates ranging from 5 to 50%, the delayed recognition of NIV failure may cause intubation delay, increasing morbidity and mortality [40].

When tracheal intubation occurs, a strategy of protective lung ventilation, especially in the coexistence of nosocomial pneumonia, combines low tidal volume (6–8 mL/kg based on predicted body weight) and high PEEP (10–15 cmH_2_O) [35]. However, in this regard, it should be considered the use of esophageal pressure monitoring in order to choose the best PEEP and optimize mechanical ventilation [41]. In obese patients, especially during the weaning phase, high transpulmonary pressure may be generated, due to excessive respiratory drive combined with vigorous inspiratory efforts and active abdominal muscle contractions, leading to a possible lung injury [42].

**Fig. 1:**
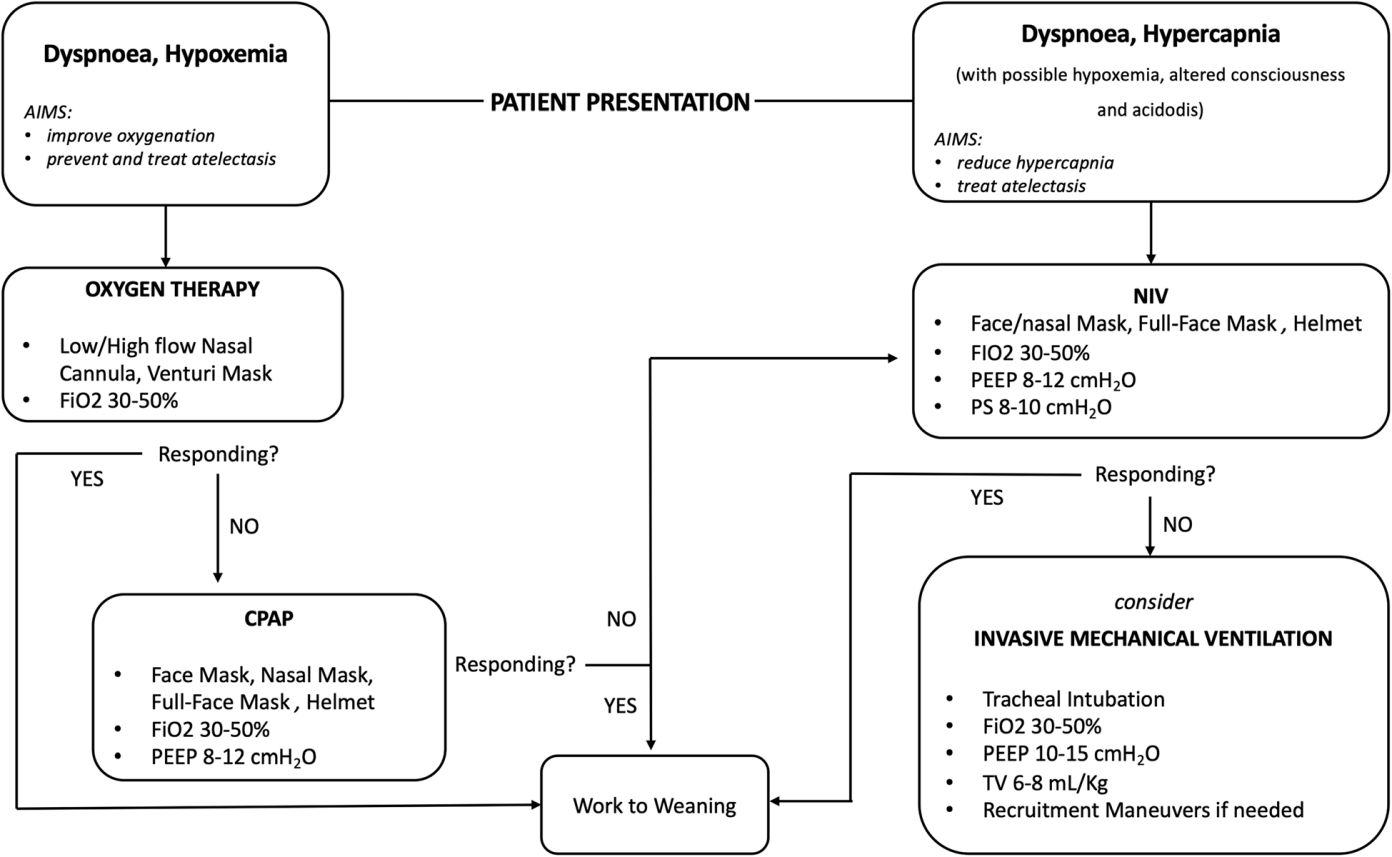
Respiratory management of acute respiratory failure in obese patients. C-PAP, continuous positive airway pressure; NIV, non-invasive mechanical ventilation; PEEP, positive end-expiratory pressure; TV, tidal volume

Respiratory management of obese patients affected by ARF is summarized in a proposed flowchart (Fig. 1).

### Trauma

Obese patients show characteristic pathophysiologic elements in the context of trauma: limb, pelvis, and thorax injuries are prevalent, with a lower incidence of abdominal and cranial traumas [32, 43].

The assessment of motor, sensory, and reflex functions is more challenging in patients with morbid obesity, because of the size and previous impaired perception of pain and reduced joint mobility and muscle strength due to the weight of the adipose tissue. The entire body surface should then be examined, especially the perineal region and lower abdomen [43].

Primary and secondary transport can be very challenging, requiring special human resources, devices, vehicles, and skills that are not always readily available [44].

A recent survey [45] pointed out the necessity to increase the training of health professionals specifically for these special patients in order to manage the most encountered critical issues: difficulties in patient extraction and transport; the absence of adequate aids, such as collars and spinal boards suitable for obese patients; and diagnostic difficulties and limitations of focused assessment with sonography in trauma (FAST) due to the presence of abundant adipose tissue.

The interaction between body mass and outcome has been incompletely explored, and the evidence of literature is contradictory. Obesity has been associated with increased mortality after motor vehicle collisions, despite comparable or reduced injury severity among obese patients [46–50]. Some have suggested that this demonstrates a “cushion” effect in automobile accidents, and the increased mortality is secondary to other systemic factors. There is some evidence that observed associations between obesity and mortality may have more to do with post-injury care than the injury itself. Morbid obesity is associated with a higher risk of post-traumatic morbidity, a higher incidence of hospitalization in intensive care units, long hospital stay, and more frequent complications such as pulmonary embolism, prolonged mechanical ventilation, infections, bedsores, wound dehiscence, acute renal failure, and multi-organ failure [49, 51–53]. Increasing BMI appears to have a protective effect on mortality in certain disease states, known as “the obesity paradox.” [54] Several studies have shown either a U-shaped or a J-shaped relationship between BMI and mortality in which overweight and class I obese patients have increased survival compared to normal weight and underweight patients, with mortality trending upwards in class II and III obesity.

In surgical critical care, obese patients represent a particularly challenging population with intra- and postoperative risk factors; as a patient’s BMI increases, their risk of developing surgical complications increases as well. In surgery, obesity resulted in a more significantly longer intervention time, higher intraoperative blood loss, and rate of surgical site infections [43, 55].

It is important to monitor blood glucose levels, which can be altered in critical patients with metabolic syndrome or insulin-dependent diabetes mellitus. Care should also be taken to control body temperature, which can easily drop to critical levels in a patient with a large body surface area. This will compromise the outcome, as it increases the risk of postoperative residual curarization, coagulation impairment, dehiscence of anastomosis or surgical wounds, infections, and decubitus ulcers.

The patients with morbid obesity are often under-resuscitated with a slower resolution of base deficit and pH and a higher mortality from persistent hemorrhagic shock due to relative hypovolemia. It has been demonstrated that an underestimation of volume requirements in the patients may be the cause of decreased cardiac output and tissue oxygenation with poor outcome [56]. The study of Nelson et al. [57] shows that the metabolic derangement in obese patients is not reflected in significantly lower systolic or mean arterial blood pressure, thereby facilitating inadvertent under-resuscitation. They emphasized the need for goal-direct therapy for the resolution of metabolic acidosis and not only for the normalization of hemodynamic parameters.

The metabolic syndrome leads to a hypercoagulable state which needs special attention to the prevention of thromboembolic events. Prevention of deep venous thrombosis can be difficult at best. Mechanical devices often do not fit patients with obesity, and a higher dose of low-molecular-weight heparins may be required to achieve therapeutic antiXA levels. Much of the existing data on venous thromboembolism (VTE) chemoprophylaxis in obese patients are from the experience of the bariatric surgery population. Due to the lack of sufficient data, however, there are no universally accepted VTE chemoprophylaxis recommendations in terms of the pharmacological agents, dosage, frequency, and duration for high-risk obese patients. The greatest consensus in the literature regards the use of enoxaparin at the dosage 0.5 mg/kg BID, which allows to achieve therapeutic antiXa levels, although it is not associated with a reduction in VTE, increasing the risk of bleeding complications [58].

Finally, obesity is associated with a pro-inflammatory state which affects post-injury inflammatory response with an increased risk of nosocomial infections and organ dysfunction. An active surveillance of biological specimens with early recognition of dangerous micro-organisms and a prudent policy of antibiotic administration are both of paramount importance to the preventive rise of multidrug-resistant infections. In motor vehicle accidents, obese people are relatively protected from abdominal and pelvic injuries because of their soft tissues. However, they are more likely to incur a pelvic ring injury, because energy absorbed by the abdomen is transferred to the pelvis. They are also more likely to fracture the peripheral structures such as the distal femur, ankle, or calcaneus and also experience degloving injury. Even with low-energy trauma, they have a tendency to experience comminuted fracture with skin and soft tissues injuries, especially the distal end of the long bones. Knee dislocations following low-energy trauma have also been described in obese people, with a high rate of neuromuscular complications, which may require amputation of the leg [59].

The treatment of arm fractures usually requires internal fixation. During orthopedic surgery, given the potential for respiratory complications in the emergence from anesthesia and in the immediate postoperative period, regional anesthesia should always be preferred over general anesthesia whenever possible [60]. However, special considerations should be done for those patients with a prediction of difficult airways. In these cases, as suggested by some authors, the decision should take into account several aspects including patient characteristics, surgical environment, clinician experience, and device availability, over a rescue strategy to manage the airway [61].

### Burns

Pathological obesity is also associated with an increase in mortality in burn patients. The most commonly used methods for determining the burn areas do not take into account BMI. Inaccurate determination of burn size leads to inadequate volume management that can generate serious complications such as hypovolemic shock or deepening of the burn wound. But on the other hand, excessive administration of fluids in obese patients, in association with cardiovascular comorbidities, carries the risk of acute pulmonary edema, abdominal and limb compartment syndrome, and prolongation of mechanical ventilation. The use of the Parkland formula for the administration of liquids, which is one of the main determinants of the survival of a burn patient, should be corrected with IBW [62, 63].

### Cardiopulmonary resuscitation (CPR)

The effectiveness of cardiopulmonary resuscitation depends on early defibrillation and quality chest compressions to restore the blood circulation [14, 15].

Cardiopulmonary resuscitation of the adult patient with obesity is not differently described from that in the normal-weight adult: a 30:2 sequence of compressions and ventilations, and compressions performed at a frequency of 100–120 bpm, with a depth of 5–6 cm at the lower half of the sternum, corresponding to the maximum diameter of the left ventricle.

These standard indications for adults obviously do not consider the size of obese patients and the distribution of the adipose tissue. The presence of adipose panniculus on the anterior and posterior chest wall, quantified by computed tomography in an average of 36.53 mm anteriorly and 50.73 mm posteriorly, could lead to reduced effectiveness of the compressions [64].

Abdominal fat causes cranial displacement of the diaphragm especially in the supine position, similar to what happens in pregnant women, and in this kind of patients, it is suggested to perform chest compressions to the upper third of the sternum [65]. Lee et al [66]showed, in a retrospective study on CT scan measurements, that the sternum point corresponds to the maximum point of ventricular diameter which in obese people is higher than the usual sternal site of the massage.

Cardiac massage in extremely obese patients is more tiring for operators, with the consequent risk of ineffective chest compressions. The guidelines therefore recommend to switch operators at shorter intervals than the standard 2 min [14]. Obese patients lying in a bed do not necessarily need to be moved onto the floor. Repositioning of obese patients may delay initiation of CPR, but also cause injuries to the patient and rescuers [67].

The use of mechanical chest compression devices might be considered although body dimensions and slope of the anterior chest wall limit usability of most devices. The upper limits include sternum height of 303 or 340 mm and chest width of 449 or 480 mm for piston devices, chest circumference of 130 cm, chest width of 380 mm, and body weight of 136 kg for devices equipped with a load-distributing band [68, 69].

Obese patients have higher transthoracic impedance caused by the adipose tissue of the chest wall, but there is no evidence in the literature of a correlation between BMI and the success rate of defibrillation at the first shock. During defibrillation, it is necessary to start with an energy level of 200 J, and the use of modern biphasic defibrillators allows to solve the problem of the increased impedance [8, 13]. Table 1 summarizes the key issues and optimal strategies regarding CPR in obese patients.

**Table 1:** Key issues and optimal strategies for CPR in obese patients

	Critical issues	Recommended strategy
Vascular access	The veins on the dorsum of the hand and the deep brachial vein may be neither visible nor palpable.	The veins of the antecubital fossa of the arm and the external jugular vein are easier to cannulate. If available, use ultrasound. Consider CVC placement or intraosseous route with a 45-mm needle.
Airway management	Manual ventilation with bag-mask should be difficult due to anatomical alterations.	Use a two-person technique for bag-mask ventilation. An experienced clinician should intubate the trachea early. Consider supraglottic devices if tracheal intubation fails or in case of difficult facial mask ventilation.
Chest compressions	Thoracic adipose tissue may reduce the effectiveness of the chest compressions.	Provide chest compressions greater than 5 cm in depth. Change rescuer performing chest compression more frequently. If applicable, consider the use of mechanical chest compression.
Cardiovascular drugs	Patients with obesity may receive an inadequate dose of emergency drugs during CPR.	*Adrenaline*—1 mg. *Amiodarone*—300 mg first time, 0.5–0.75 mg/kg second time. *Lidocaine*—1–1.5 mg/kg according to IBW. *Magnesium sulfate*—2 g, repeat after 10–15 min. *Calcium chloride* 10%—1 g. *Atropine*—0.5 mg, repeat to a maximum dose of 3 mg. *Isoprenaline*—5 μg/min. *Adrenaline* (c.i.)—2–10 μg/min
Defibrillation	Higher transthoracic impedance caused by thoracic fat.	Start with an energy level of 200 J

*IBW* ideal body weight, *CVC* central venous catheter, *c.i* continuous infusion

### Drug dosage during CPR

Wang et al. [70] found that patients with a body weight greater than 82.5 kg may receive an inadequate dose of adrenaline compared with the standards indicated by the guidelines. Gough and Nolan [71] showed, however, that high doses of adrenaline (> 1 mg) are associated with an increase in return of spontaneous circulation (ROSC) and survival but also worsening of neurological outcomes after cardiac arrest.

The drugs administered to obese patients during an emergency setting show important pharmacokinetic and pharmacodynamic alterations with respect to normal-weight individuals (volume of distribution, protein binding, renal clearance, and hepatic metabolism). As a result, doses need to be adjusted according to IBW, LBW, adjusted body weight (ABW), or total body weight (TBW) (Table 2) [44, 72–74].

**Table 2:** Emergency drug dosing in obese patients. Adapted from Cataldo et al. [72]

Drugs	Loading dose	Maintenance dose
*Neuromuscular blockers and antagonists*		
Succynilcholine	TBW	–
Vecuronium	IBW	IBW
Atracurium	LBW	LBW
Rocuronium	LBW	LBW
Sugammadex	TBW	–
Neostigmine	ABW	–
*Sedative hypnotics*		
Benzodiazepine	IBW	IBW
Propofol	LBW	ABW
Thiopental	LBW	IBW
Phenobarbital	TBW	IBW
Ketamine	TBW	IBW
Etomidate	TBW	–
Dexmedetomidine	–	LBW
*Analgesics*		
Morphine	LBW	–
Remifentanil	LBW	–
Fentanil	LBW	–
Sufentanil	LBW	–
Alfentanil	ABW	–
Paracetamol	LBW	–
*Corticosteroids*		
Methylprednisolone	IBW	IBW
*Anti-epileptics*		
Phenytoin	IBW + [1.33 × (TBW − IBW)]	IBW
Valproic acid	–	IBW
Carbamazepine	–	IBW
*Beta-blockers*		
Propranolol	IBW	IBW
Labetalol	IBW	IBW
Metoprolol	IBW	IBW
Esmolol	IBW	IBW
*Calcium channel blockers*		
Verapamil	TBW	IBW
Diltiazem	TBW	Titration
*Antiarrhythmics*		
Lidocaine	ABW	ABW
Procainamide	IBW	IBW
Amiodarone	IBW	IBW
Digoxin	IBW	IBW
Adenosine	IBW	IBW
*Catecholamines*		
Dobutamine Dopamine Epinephrine Norepinephrine Phenylephrine Vasopressin Milrinone		There are no clinical studies in obese patients. According to the literature, ABW or IBW could be used to avoid overdoses, titrating the dose as a function of the clinical target. ABW in patients ≤120 kg

IBW for men: height (cm) – 100; IBW for women: height (cm) – 110

*IBW* ideal body weight, *TBW* total body weight, *ABW* adjusted body weight, *LBW* lean body weight

ABW = IBW + 40% TBW

LBW = in men approximately 90 kg, in women approximately 70 kg

More studies are needed to optimize cardiopulmonary resuscitation of the obese patient in order to point out specific guidelines for life support, as it happens for other particular patient populations, like the pediatric one [75].

### Post-resuscitation care

Once spontaneous circulation is restored, post-resuscitation care begins, consisting of the identification and treatment of the causes that led to the cardiorespiratory arrest along with the assessment and treatment of ischemia-reperfusion injury.

In the meantime, hemodynamic stability [mean arterial pressure (MAP) ≥ 65 mmHg], good oxygenation and normocapnia, glycemic control, and EEG monitoring must be guaranteed [76].

Some studies have investigated therapeutic hypothermia during coma after out-of-hospital cardiac arrest with a shockable rhythm, keeping the body temperature between 32 and 36 °C for 12–24 h [77].

However, the use of therapeutic hypothermia is controversial, and a recent evidence suggests its non-superiority with respect to normothermia in reducing death incidence at 6 months [78].

Moreover, cooling times are longer in patients with obesity than normal-weight patients [79] and variable depending on the devices used (external or endovascular), and the clinical results are conflicting: some investigators claim a lower mortality and better neurological outcomes [80], while others maintain that a BMI ≥ 30 kg/m^2^ is a risk factor for post-hypothermic mortality [81].

Various factors must be considered in post-resuscitation care, including comorbidities, the cause of the cardiac arrest, the patient’s age and sex, the duration of CPR, the concept of the obesity paradox, and the cytokine structure of the obese patients, which predisposes these patients to different ischemia-reperfusion injury. Further randomized clinical trials are needed to obtain more clear-cut results [82].

### Special considerations on resuscitation during the COVID-19 pandemic

During the COVID-19 pandemic, the Centers for Disease Control and Prevention (CDC) reported in their Morbidity and Mortality Weekly Report of April 17, 2020, that patients hospitalized with COVID-19 had one or more underlying conditions, the most common being hypertension (49.7%), obesity (48.3%), chronic lung disease (34.6%), diabetes mellitus (28.3), and cardiovascular disease (27.8%) [83–86]. A report on 4103 patients with COVID-19 in New York City found that the most important clinical features leading to hospital admission were age > 65 years and obesity [87–89]. In Italy, 10.7% of patients who died of COVID-19 were affected by obesity [90]. Obesity was the most common comorbidity described during the Italian outbreak [91]. Obese patients are also at higher risk of severe complications of COVID-19 [92, 93] by virtue of the obesity-driven increased risk of chronic disease [94]. Moreover, obese patients may develop atelectasis and lung derecruitment, making oxygenation and intubation complicated, hence putting these patients in the prone position is difficult and risky [95–97].

Recent European Resuscitation Council COVID-19 Guidelines [98] focus to reduce the risk of airborne spread of the virus and to prevent rescuers during CPR, chest compressions, and airway management [99]. But the global pandemic highlighted that appropriate resources for patients with (severe) obesity are often inadequate: for example, access to imaging may be limited by a lack of machines able to accommodate them or by an insufficient number of bariatric beds [100]. Challenges to the healthcare systems include more difficult intubations, more complex lifting and handling demands, and more difficulty to obtain an imaging diagnosis even with pulmonary ultrasound. People with severe obesity may therefore be seriously disadvantaged regarding health care in the COVID-19 era [101, 102].

## Conclusion

Even prior to the development of critical illness, obese patients have alterations in respiratory physiology, circulatory physiology, and pharmacokinetics that significantly affect their emergency treatment and resuscitation. Emergency management in critically obese patients is an intellectual, procedural, and technical challenge. Organization and anticipation based on the understanding of the physiopathology related to obesity are very important for the physician to be mentally and physically ready to face the associated issues. Further studies are expected to investigate new techniques and devices capable to improve the efficacy of cardiopulmonary resuscitation and airway management in obese patients, leading to specific guidelines for this particular population.
